# *Cynara cardunculus* L. var. *scolymus* L. Landrace “Carciofo Ortano” as a Source of Bioactive Compounds

**DOI:** 10.3390/plants13060761

**Published:** 2024-03-07

**Authors:** Valentina Laghezza Masci, Enrica Alicandri, Chiara Antonelli, Anna Rita Paolacci, Rosita Marabottini, William Tomassi, Giuseppe Scarascia Mugnozza, Antonio Tiezzi, Stefania Garzoli, Vittorio Vinciguerra, Anna Maria Vettraino, Elisa Ovidi, Mario Ciaffi

**Affiliations:** 1 Department for the Innovation in Biological, Agrofood and Forestal Systems, Tuscia University, 01100 Viterbo, Italy; laghezzamasci@unitus.it (V.L.M.); enrica.alicandri@unitus.it (E.A.); chiara.antonelli@unitus.it (C.A.); arpaolacci@unitus.it (A.R.P.); marabottini@unitus.it (R.M.); william.tomassi@outlook.com (W.T.); gscaras@unitus.it (G.S.M.); antoniot@unitus.it (A.T.); vincigue@unitus.it (V.V.); vettrain@unitus.it (A.M.V.); eovidi@unitus.it (E.O.); 2 Department of Drug Chemistry and Technology, Sapienza University, 00185 Rome, Italy; stefania.garzoli@uniroma1.it

**Keywords:** *Cynara cardunculus* L. var. *scolymus*, “Carciofo Ortano” landrace, artichoke waste, artichoke leaf extracts, phenolic composition, volatile organic compounds (VOCs), secondary metabolites, cytotoxicity, *Alternaria alternata*

## Abstract

The preservation of agricultural biodiversity and socioeconomic development are relevant both to enhance domestic production and to support innovation. In the search for new biomolecules, we have focused on the “Carciofo Ortano” landrace, growth in the northern part of the Lazio region. Artichoke cultivation generates substantial by-products, including leaves, stems, and roots, which could serve as valuable sources of biomolecules and prebiotic dietary fiber. To valorize the leaf waste of the “Carciofo Ortano” landrace, a multidisciplinary approach was applied. Chemical analysis using HPLC-DAD identified mono-O- and di-O-caffeoylquinic acids and the sesquiterpene cynaropicrin in all artichoke leaf extracts. SPME-GC/MS analyses detected aliphatic alcohols in the fresh leaf samples. Antiproliferative and cytotoxic studies on cancer (SH-SY5Y, MCF-7, MDA) and normal (MCF-10A) human cell lines revealed that leaf extracts induced a selective dose and time-dependent biological effect. While showing slight activity against environmental bacterial strains, artichoke leaf extracts exhibited significant antifungal activity against the phytopathogenic fungus *Alternaria alternata*. Overall, the results highlight the potential of “Carciofo Ortano” cultivation by-products as a rich source of biomolecules with versatile applications in humans, animals, and the environment.

## 1. Introduction

Globe artichoke (*Cynara cardunculus* L. var. *scolymus* (L.) Fiori) is a herbaceous perennial plant native to the Mediterranean basin [1]. The predominant commercial utilization of this plant involves the consumption of its edible immature inflorescence, commonly referred to as the “capitulum” or head, in various forms such as raw, boiled, steamed, fried, and as an ingredient in diverse dishes, as it combines good sensory and health features [2,3]. Indeed, the artichoke is an important component of the Mediterranean diet and of the food pyramid. The high nutritional value of artichoke heads can be ascribed to their low lipid content and high levels of fibers (including inulin) as well as minerals, vitamins, and numerous bioactive secondary metabolites [4,5,6]. Specifically, artichoke heads are recognized for being a rich source of hydroxycinnamic acids (predominantly mono- and di-O-caffeoylquinic acids), flavone glycosides (primarily derivatives of luteolin and apigenin), and terpenes (mainly sesquiterpene lactones) [4,7]. These bioactive compounds contribute to the health-promoting effects on humans, as evidenced by various studies highlighting the potential use of the artichoke as an antilipidemic, diuretic, anti-inflammatory, and hepatoprotective agent [4,5,7]. As a result, the globe artichoke is considered a “functional food” with significant pharmacological and nutraceutical activities [6,7,8].

Globally, in 2022, the cultivated area for the artichoke spans approximately 113,000 hectares, resulting in a total production of about 1.58 million tons, with 0.61 million tons originating from Europe [9]. Italy, being the foremost artichoke-producing country in the world, contributes significantly with a cultivated area of about 38,000 hectares and a total flower head production of around 378,000 tons [9].

In general, the artichoke exhibits a notably low harvest index [10], with approximately 80–85% of the total plant biomass comprising artichoke crop residues [11]. The processing of artichokes on an industrial scale results in a substantial volume of biomass waste, primarily composed of bracts and stems discarded during the harvesting process [12]. Nonetheless, according to Gominho et al. [11], this waste constitutes a relatively small proportion (15–30% dry weight) of the overall biomass. The residual portion (70–85% dry weight) comprises leaves and stalks, which persist in the field when the artichoke is cultivated as a perennial crop without further utilization. These agricultural residues possess elevated moisture contents, rendering them susceptible to microbial proliferation and potential environmental contamination. Nevertheless, these by-products are rich in various phytochemicals, akin to those found in the edible flower heads of artichokes, including phenolic and terpenoid compounds [7,13,14,15], inulin [7,16], and dietary fiber [7,17]. Remarkably, the phenolic content of these residues is higher than that of other phenol sources such as carrot peels, grape pomace, and spent coffee grounds [12]. Several epidemiological and pharmacological studies have demonstrated the health-promoting effects of globe artichoke extracts from non-edible plant parts, including hypolipidemic, hepatoprotective, anti-obesity, antioxidant, and anti-inflammatory activity [4,7]. Significant antibacterial and antifungal effects are also recognized [7]. For this reason, in addition to the culinary use of the budding flower-head, the abundant leaf biomass of this species has gained importance as a raw material to produce nutraceuticals, food preservatives, drugs and cosmetics [3,4,7], opening new possibilities for the valorization of artichoke crop residues. Previous studies also indicated that the quantity and quality of the bioactive compounds (mainly polyphenols) present in different non-edible plant parts depend on preharvest factors such as the genotype, growth conditions, and phenological stage [13,18,19,20,21].

The use of plant-derived substances as anti-cancer agents is an emerging aspect in the approach to cancer treatment. This trend is driven by their accessibility, affordability, and remarkable efficacy, while presenting minimal side effects compared to synthetic drugs [22]. Cancer cells display several hallmarks, including uncontrolled division, altered signaling pathways, stress responses, angiogenesis, metastasis, and immune evasion [23]. Cancer chemoprevention may be a promising strategy for blocking, inhibiting, reversing, or delaying the process of carcinogenesis, and the use of botanical supplements offers a convenient approach for the administration of potentially chemopreventive agents [24]. Some studies have explored the potential anti-cancer effects of artichoke and its role in cancer prevention, attributing these effects to its antioxidant and anti-inflammatory properties, but further research is needed [25].

Throughout human history, plant diseases have had an intensive and detrimental impact on both human society and the forest and agricultural environment [26,27,28,29]. In recent years, there has been growing interest in sustainable and eco-friendly methods to control plant diseases, with plant extracts emerging as a promising solution. Although there are several studies about the possibility of using plant extracts in management of plant diseases, currently, little information is available on the use of artichoke leaf extracts [30,31,32,33,34].

This study highlights a landrace known as “Carciofo Ortano”, belonging to the “Romanesco” varietal type as defined by Alicandri et al. [35], which focused on the molecular, morphological, and nutritional characterization of this landrace. It is cultivated in the Lazio region, and it originated from the municipality of Orte in the province of Viterbo and continues to be grown along the riverbanks of the Tiber River. A small number of dedicated farmers preserve the cultivation of “Carciofo Ortano” primarily for personal use, maintaining a tradition passed down through generations. However, the survival of this landrace is threatened by factors such as the aging population of custodians, limited cultivation areas mostly confined to local vegetable gardens, and the introduction of commercial varieties [35].

From the perspective of safeguarding agricultural biodiversity and advancing a circular economy paradigm, the aim of the present study was to assess the added value of the by-products of the “Carciofo Ortano” that are discarded during the local routine agricultural practices. Consequently, the investigation focused on the analysis of bioactive constituents (phenolic and volatile compounds) present in leaves collected after the harvesting period from representative genotypes of this landrace, namely Orte 1 and Orte 2, in comparison to the “Grato 1” clone, used as a reference genotype. The assessment extended to investigating the antiproliferative and cytotoxic effects on human neuroblastoma and adenocarcinoma breast cells of leaf extracts, as well as their antibacterial activity. Additionally, the efficacy of artichoke leaf extracts against *Alternaria alternata*, a prevalent pathogen affecting various vegetables, ornamental and forest plants, was examined.

## 2. Results

### 2.1. Phenolic Composition of Leaf Extracts

In this study, a total of 11 hydroxycinnamate and flavone compounds, along with one sesquiterpene, were identified based on the chromatographic retention time and UV spectrum profile (Figure S1 and Table S1). While the qualitative composition was similar across all samples, notable quantitative differences were observed. The total polyphenol content exhibited an ascending pattern, increasing from 6882 mg/kg DW in Grato 1 to 10,366 mg/kg DW in Orte 1, and further to 16,814 mg/kg DW in Orte 2 (Table 1). This rise can be attributed to the significant contribution of mono-O- and di-O-caffeoylquinic acids, particularly 5-O-caffeoylquinic acid (chlorogenic acid), 3,5- and 1,5-di-O-caffeoylquinic acids. Notably, the levels of these compounds increased substantially from Grato 1 to Orte 2 (Grato 1 < Orte 1 < Orte 2). Minimal quantities of 1,3-di-O-caffeoylquinic acid (cynarin) were detected in all samples, with an apparent undetectable presence in Orte 1 (Table 1).

Conversely, the total content of flavones exhibited an opposite trend (Grato1 > Orte 1 > Orte 2), with values ranging from 3405 mg/kg DW in Grato 1 to 2586 mg/kg DW in Orte 1, and further to 1429 mg/kg DW in Orte 2 (Table 1). Glycosylated luteolin, in both 7-O-rutinoside and 7-O-glucoside forms, contributed predominantly to the content of flavones to a similar extent across all samples.

Finally, HPLC analysis revealed a substantial presence of sesquiterpene cynaropicrin in all three leaf samples, with higher levels in Orte 2 (8143 mg/kg DW) compared to Grato 1 (4994 mg/kg DW) and Orte 1 (3946 mg/kg DW) (Table 1).

### 2.2. Volatile Organic Compounds (VOCs)

The SPME chromatographic analyses allowed the identification of volatile components belonging to different chemical classes such as aliphatic alcohols, monoterpenes and sesquiterpenes (Table 2). In detail, 3-methyl-1-butanol and 2-methyl-1-butanol were found in all analyzed leaf samples. In particular, 2-methyl-1-butanol was detected in the leaf samples of the three genotypes with almost superimposable average percentage values. In contrast, 3-methyl-1-butanol emerged as the predominant compound in Grato 1 (73.4%) and Orte 2 (71.4%), with a comparatively lower content in Orte 1 (39.9%). The leaves of Grato 1 were distinguished by the exclusive presence of these two compounds, whereas those of Orte 2 also featured minor quantities of β-eudesmene (0.1%) and β-caryophyllene (0.1%). Furthermore, the leaves of Orte 1 were characterized by the additional presence of 3-hexen-1-ol (39.1%).

### 2.3. Antiproliferative and Cytotoxic Activities

To evaluate the antiproliferative activity and cytotoxic effect of leaf extract of Orte 1, Orte 2 and Grato 1 genotypes, MTT assays were performed in a dose- and time-dependent manner on human cancer cell lines (SH-SY5Y, MCF7 and MDA cell lines) and the human normal cell line (MCF10A cell line).

The antiproliferative activity of the extracts was studied by measuring the cell viability of cancer and normal cells after exposure for 24 h, 48 h, and 72 h. As shown in Table 3, the leaf extracts demonstrated dose- and time-dependent anti-proliferative activity on MCF7 and MDA cells. The EC_50_ values of Orte 1 leaf extract against MCF7 and MDA cells decreased significantly by 50% with an increasing treatment time from 24 h to 48 h (85.2 ± 2.3 μg/mL to 44.7 ± 4.2 μg/mL for MCF-7 and 69.0 ± 2.3 μg/mL to 33.4 ± 0.9 μg/mL for MDA), and, particularly, a more significant inhibition of growth was observed at 72 h, with a decrease in EC_50_ values to 23.3 ± 1.2 μg/mL and 26.5 ± 1.2 μg/mL for MCF7 and MDA, respectively. No time-dependent effect was obtained for Orte 1 leaf extract on SH-SY5Y cells, as evidenced by the EC_50_ values of 18.3 ± 0.2 μg/mL, 12.6 ± 0.5 μg/mL and 35.4 ± 1.2 μg/mL at 24 h, 48 h and 72 h respectively.

As regards the Orte 2 leaf extract, a progressive reduction in EC_50_ values in MCF7 cells was observed from >250 μg/mL at 24 h to 33.6 ± 1.6 to μg/mL at 72 h. A slighter trend was also observed for MDA cells with EC_50_ of 75.6 ± 1.8 μg/mL to 40.1 ± 2.9 μg/mL at 24 h and 72 h, respectively. Like Orte 1, the antiproliferative activity of Orte 2 leaf extract on SH-SY5Y cells was not correlated with the treatment exposure time (51.8 ± 5.3 μg/mL, 47.1 ± 9.1 μg/mL and 59.8 ± 3.6 μg/mL at 24 h, 48 h and 72 h, respectively).

The antiproliferative activity was observed on MDA cell lines with EC_50_ values ranging from 49.2 ± 0.5 μg/mL to 17.7 ± 0.4 μg/mL at 24 h and 72 h for Grato 1 leaf extract.

**Figure 1:**
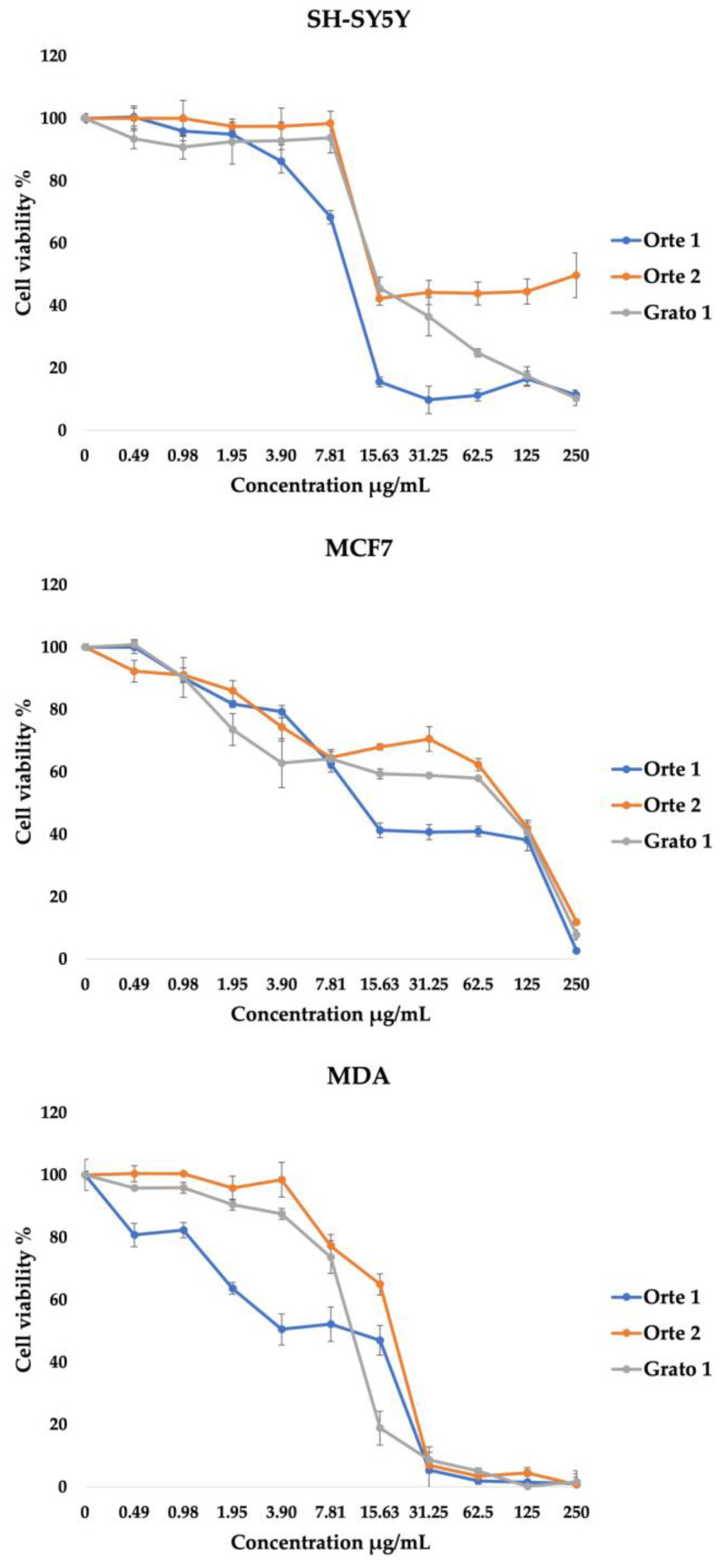
Line graphs of the dose–response after 72 h of treatment of Orte 1, Orte 2 and Grato 1 leaf extracts in the three cancer cell lines considered (SH-SY5Y, MCF7 and MDA) (*p* < 0.05).

The cytotoxicity effect of the extracts was assessed by examining the cell viability at 72 h. The results showed that leaf extracts of all three genotypes induced a potent dose-dependent cytotoxic effect on the considered cancer cell lines (Figure 1). Orte 1 and Orte 2 leaf extracts showed good cytotoxicity with low EC_50_ values ranging from 23.3 ± 1.2 μg/mL on MCF7 cells for Orte 1 to 59.8 ± 3.6 μg/mL on SH-SY5Y cells for Orte 2. With a lower EC_50_ value, the leaf extract of the Grato 1 plants was more cytotoxic against MDA cells (17.7 ± 0.4 μg/mL).

Furthermore, all the extracts studied showed no antiproliferative activity or cytotoxic effect on normal cell line (MCF10A), indicating that these extracts are selective toward human cancer cells with minimal toxicity on normal human cells (Table 3).

### 2.4. Antimicrobial Activity

The antibacterial activity of the leaf extracts of Orte 1, Orte 2, and Grato 1 was evaluated by measuring the minimum growth inhibition concentration (MIC) and the minimum bactericidal concentration (MBC) on five different environmental bacterial strains, both Gram-positive (*Bacillus cereus*, *Kocuria marina* and *Acinetobacter bohemicus*) and Gram-negative (*Escherichia coli* and *Pseudomonas fluorescens*).

The leaf extracts tested showed activity only on *B. cereus* and *A. bohemicus* strains (Table 4). Concerning Orte 1 extract, the bacterial growth inhibition activity was observed at a concentration of 0.5 mg/mL on *B. cereus*, a concentration that increased to 2 mg/mL on *A. bohemicus*. In addition, in the case of Orte 2 extract, as with Grato 1, the antimicrobial activity was found only against *B. cereus* and *A. bohemicus*; in both cases and for both genotypes, the MIC values were 2 mg/mL (Table 4).

The exposure with Orte 2 leaf extracts provided MBC values equal to the MIC in *B. cereus* and *A. bohemicus* while the MBC values for the extracts of Orte 1 and Grato 1 were doubled compared to the MIC values obtained (Table 4).

### 2.5. Antifungal Activity

**Figure 2:**
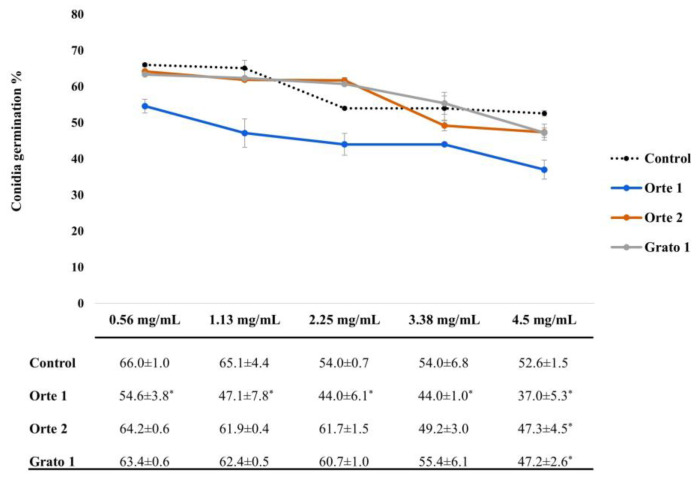
Effect of different doses of Orte 1, Orte 2 and Grato 1 leaf extracts on the germination of *A. alternata* Mn2 conidia. The values are expressed as mean value ± SD. Asterisks (*) mean a significant difference between treatments and control (*p* < 0.05).

DMSO proved its neutral effect on the growth of *A. alternata* Mn2, as it did not decrease the percentage of conidia germination at a concentration of 2%. The efficacy of the leaf extracts of the three genotypes on the reduction in conidia germination was significantly affected by concentration (0.5625, 1.125, 2.25, 3.375 and 4.5 mg/mL) (Figure 2).

As illustrated in Figure 2, Orte 1, Orte 2 and Grato 1 leaf extracts significantly reduced the germination of *A. alternata* Mn2 conidia by 8, 18 and 8% (ANOVA, *p* < 0.05), respectively, at a maximum dose of 4.5 mg/mL. Conversely, Orte 1 leaf extract exhibited a significant effect on the reduction in the percentage of conidia germination even at lower concentrations, namely 0.56, 1.13, 2.25, and 3.38 mg/mL (ANOVA, *p* < 0.05) (Figure 2).

## 3. Discussion

The artichoke “Carciofo Ortano’’, a landrace at risk of genetic erosion, is still cultivated in the plains along the Tiber River in the countryside of the Orte municipality in the Lazio region (Central Italy). It is highly appreciated for the nutritional and organoleptic values of its immature edible flower heads [35]. Its cultivation and valorization contribute to and reinforce the agroeconomic and social development of the local territory. However, the cultivation of artichokes results in a substantial amount of waste, from stems, leaves, and outer bracts, posing a significant concern for waste management. This study outlines an approach aimed at converting bio-waste into by-products with marketable properties. Specifically, the biological and antimicrobial activities of phenolic-rich extracts from leaves of two representative genotypes of “Carciofo Ortano” (Orte 1 and Orte 2) and the Grato 1 clone have been determined.

### 3.1. Chemical Composition

The quantitative and qualitative polyphenol profile in artichoke leaf extracts is extensively documented in the literature, indicating that it depends on various factors, including the extraction and determination methodologies, genotype, environmental conditions, agronomic practices, and specific physiological stage of the analyzed leaf material [13,15,36,37,38,39].

In this study, the total phenolic content, calculated as the sum of individual phenolic compounds, differed significantly among the three analyzed genotypes, ranging from 6.882 to 16.814 g/kg DW, with the two “Carciofo Ortano” genotypes showing the highest values. Pandino and coworkers [13], by analyzing several clones selected from the two Sicilian landraces “Spinoso di Palermo” and “Violetto di Sicilia”, reported lower values in the range of 2.195–8.466 g/kg DW compared to those obtained in this study, in particular for the “Carciofo Ortano” genotypes. These differences could be attributed to the diverse investigated genotypes and to the different extraction conditions used for the recovery of the phenolic compounds. In our investigation, an ultrasound-assisted method was employed, in contrast to conventional methodologies reliant on shaking plant material with alcoholic solvents. Ultrasounds typically enhance the extraction of bioactive compounds by leveraging the cavitational effect, thereby promoting the liberation of extractable compounds and augmenting mass transport through diffusion or by disrupting plant cell walls [37]. On the other hand, Bonasia et al. [39] documented higher values for the phenolic content, as the sum of clorogenic acid, cynarin, luteolin and apigenin, in artichoke leaf extracts, ranging from 11.7 to 22.8 g/kg DW, within three seed propagated hybrids (“Tempo”, “Opal” and “Madrigal”) and two landraces from the Puglia region (“Violetto di Foggia” and “Brindisino”). Apart from the different genotypes analyzed, the observed differences can likely be attributed to the age and positioning of the leaf material analysed in our study, where a bulk of leaf biomass was deliberately sampled from artichoke plants after the harvesting of flower heads, in contrast to the referenced study, which focused on the 2–3 leaves borne by flower stems. As evidence of these differences, El Senousy et al. [40] documented that the top-positioned leaves of the artichoke plant represent a better source of polyphenol compounds compared to the basal ones. Despite the broader range of variations reported in the literature regarding the phenolic content of artichoke by-products, the level of total phenolic compounds found in the leaf waste of “Carciofo Ortano” underscores its potential as a cost-effective and promising reservoir of bioactive compounds.

In accordance with information available in the literature [17,41,42,43], our study identified caffeoylquinic acids as the predominant phenolic compounds in artichoke leaf extracts. However, significant variations were observed among the three examined genotypes, with the proportions for the phenolic acids representing 91.5%, 75.0%, and 52.7% of the total phenolic compounds in Orte 2, Orte 1, and Grato 1 leaf extracts, respectively. Conversely, significantly lower relative amounts of caffeoylquinic acids, ranging from 0 to 46.2%, were reported in the previously mentioned study of Pandino et al. [13], in which leaf samples from different clones of the “Spinoso di Palermo” and “Violetto di Sicilia” landraces were considered.

Among caffeoylquinic acids, 5-O-caffeoylquinic acid (chlorogenic acid) and 1,5-di-O-caffeoylquinic acid were the most abundant in the three genotypes analysed. In particular, the content of the chlorogenic acid detected in the leaves of the two “Carciofo Ortano” genotypes (5.156 and 7.842 g/kg DW) was relatively high compared to the data reported in the literature for different globe artichoke cultivars, such as “Violetto di Sicilia” (0.1–1.9 g/kg DW) and “Spinoso di Palermo” (0.7–2.1 g/kg DW) [13], Blanca de Tudela (2.4 g/kg DW) [36], Madrigal (2.8 g/kg DW) [43], “Tondo di Paestum” (2.77 g/kg DM) and “Bianco di Pertosa” (4.91 g/kg DM) [42]. As previously discussed, this comparative analysis with literature data fails to consider variations in extraction conditions, which could exert a significant impact on the resulting yield. In contrast, cynarin (1,3-di-O-caffeoylquinic acid) and caffeic acid were found in very low concentrations in the leaves of the three genotypes analysed, matching with the data reported in the literature [37,39,42]. Despite their relatively low content, these compounds stand out as prominent caffeoylquinic acids within artichoke leaf extracts, owing to their documented potential health benefits. These include choleretic and cholesterol-lowering properties, hepato-protective and anti-atherosclerotic activities, antioxidative properties, and anti-carcinogenic effects [4,44,45,46].

Regarding flavones, the predominant compounds were luteolin-7-glucoside and luteolin-7-rutinoside, while negligible quantities of other flavones were found in our leaf extracts. Consistent with our findings, several studies indicated that luteolin derivatives are more abundant in artichoke by-products than in edible part of this vegetable, where the most prevalent flavones appear to be the apigenin derivatives, especially apigenin-7-O-glucuronide [13,47,48]. It is worth highlighting the contrasting pattern observed in the cumulative levels of total flavones as opposed to the total quantity of caffeoylquinic acids and their individual components across the three examined genotypes. Specifically, Grato 1 showed the highest total flavones content at 3.251 g/kg DW, followed by Orte 1 at 2.586 g/kg DW and Orte 2 at 1.429 g/kg DW.

In contrast to polyphenols, little information has been reported to date regarding the quantity of cynaropicrin in artichoke leaf extracts, despite its recognized and numerous health benefits [3,49]. Rouphael et al. [15] observed a considerable range of variation for this sesquiterpene lactone (ranging from 0.045 to 12.74 g/kg FW) by analyzing leaf extracts from 19 representative artichoke cultivars grown in Europe. However, due to variations in extraction techniques, determination methods (UHPLC/Q-TOF-MS), and quantification based on fresh weight, a direct comparison between our data (ranging from 3.946 to 8.143 g/kg DW) and the findings reported in the referenced study was deemed unfeasible.

Regarding volatile compounds, in this study, we applied, for the first time, the SPME sampling technique on the untreated matrix to comprehensively elucidate the volatile profile of artichoke leaves. This methodological choice was motivated by the objective of achieving a characterization of the volatile fraction that closely mirrors the natural state of the sample, devoid of any destructive effects caused by the utilization of organic solvents.

Among the volatile compounds identified in artichoke leaves, 2-methyl-1-butanol emerged as one of the predominant compounds, displaying nearly identical percentage values across all three genotypes. Notably, this compound was found as the primary constituent in the n-butanol root extracts of *Plantago lanceolata* L., demonstrating a cytotoxic effect against HCT-116 cell lines [50]. On the other hand, 3-hexen-1-ol, one of the most important volatile organic compounds in plants, was detected only in the Orte 1 genotype. This compound has been found in fresh tea leaves as an important allelochemical in response to mechanical injury [51]. Moreover, it stands out as a substantially significant active constituent, contributing to the characteristic “green, grassy, and fresh” aroma in plant-based foods [52].

### 3.2. Biological Activities

The antimicrobial and antiproliferative activities, as well as the cytotoxic activities of artichoke leaf extracts, are well known. However, gaining a clear understanding of the significance of these activities is challenging. Various factors, such as the genotype of the plant source, variations in extraction protocols for phenolic-rich compounds, and differences in extract concentrations, contribute to substantial heterogeneity in the products employed across different studies. This diversity makes it challenging to draw comparisons among results.

#### 3.2.1. Cytotoxic Activity

Artichoke leaf extracts are characterized by the presence of numerous bioactive compounds, particularly polyphenols, which appear very promising in the prevention and management of specific cancer types. Recent studies conducted both in vitro and in vivo conditions demonstrated the ability of artichoke extracts from different plant parts to inhibit the angiogenesis and/or the proliferation of some cancer cell lines [25]. For instance, it was demonstrated that artichoke leaf extracts were able to induce a dose-dependent reduction in cell viability and inhibition of the growth of the human leukemia cell line K562 [53], HepG2 cells in hepatocellular carcinoma [31], HT-29 and HCT116 cell lines in colon cancer [54], and MCF7 and MDA cell lines in breast cancer [55,56]. In the present study, the potential cytotoxic activity of artichoke leaf extracts was performed by analyzing the cell viability on human cancer cell lines (SH-SY5Y, MCF7 and MDA) and on normal human cell lines (MCF10A) after treatment carried out in a dose- and time-dependent manner. The results highlighted that all extracts studied showed antiproliferative activity and cytotoxic effects on all the cancer cell lines considered, while no effect was found on normal cell lines, indicating selectivity toward human cancer cells.

The observed antiproliferative and cytotoxic effects of artichoke leaf extracts are suggested to be attributed to the induction of cell cycle arrest and apoptosis, evidenced by increased caspase activity and DNA fragmentation [31], disruption of the mitochondrial membrane potential and activation of caspase-dependent apoptotic pathways [55,56], and potentially by the modulation of crucial signaling pathways involved in cell survival and proliferation [54]. In addition, the ability to modulate several signaling pathways involved in cancer progression was demonstrated, as reported for artichoke leaf extracts that were found to downregulate the expression of genes associated with inflammation and cell proliferation in a colon cancer model [57].

#### 3.2.2. Antimicrobial Activity

Several studies reported that artichoke leaf extracts demonstrated noteworthy inhibitory activities against the growth of several bacteria capable of human infections, including *Staphylococcus aureus*, *Escherichia coli*, *Salmonella typhimurium* and *Bacillus cereus* [58,59].

Conversely, the antimicrobial activity obtained in this study reveals modest activity on *B. cereus* and *A. bohemicus* for all three genotypes studied, with a slightly greater effect for the Orte 1 leaf extract on *B. cereus* with an MIC of 0.5 mg/mL. Similarly, Koubaa et al. [60] observed that the compounds extracted from *C. cardunculus*, including chlorogenic acid, cynarin, luteolin-7-O-rutinoside, and cymaroside, displayed greater efficacy against fungi compared to bacteria, demonstrating a higher level of activity [60]. However, Scavo et al. [61] noted that the quantity of polyphenols does not necessarily correspond to a higher level of antibacterial activity.

#### 3.2.3. Antifungal Activity against Plant Pathogens

Several phenolic-rich plant extracts have shown promising results in controlling plant pathogens. For instance, phenolic extracts obtained from green and brown seaweeds at a dose of 1.5 g/L completely inhibited the mycelial growth and conidial germination of *M. fructicola*, *B. cinerea*, and *A. alternata*, known to cause severe pre- and post-harvest diseases, achieving a 100% inhibition rate [62]. Additionally, soybean and pomegranate extracts have demonstrated robust fungicidal activity against various plant pathogens such as *Monilinia laxa*, *B. cinerea*, and *Penicillium digitatum* [63,64].

Our study’s findings are consistent with those from the literature, indicating that phenolic compounds from artichoke leaves can inhibit the growth of plant pathogens. In particular, results showed a higher efficacy of Orte 1 leaf extract in inhibiting conidia germination of *A. alternata* Mn2 compared to Orte 2 and Grato 1 leaf extracts. According to the authors’ knowledge, this study is the first to report on the antifungal activity of artichoke leaf extracts against *A. alternata*.

### 3.3. Compounds Likely Involved in Biological Activities

Although the polyphenol-rich foliar extracts from the three examined artichoke genotypes in this study exhibited significant biological activity against both cancer cell lines and the plant pathogen *A. alternata*, variations in their antiproliferative, cytotoxic and antifungal effects may be attributable to their different polyphenol compositions rather than the overall quantity. In particular, the leaf extracts of Orte 1 and Grato 1 genotypes, although possessing a significantly lower total amount of polyphenols than Orte 2, because of a lower concentration of caffeoylquinic acids, are characterized by a greater abundance of flavones, notably luteolin derivatives. Moreover, the observed biological activities are probably not related to a single compound, but rather to several active compounds acting synergically, and some of them, which have been shown to have antitumor and antimicrobial effects, were found in significant concentration in the artichoke leaf extracts.

Among those compounds with potential synergistic action are glycosylated luteolin derivatives (luteolin 7-O-rutinoside and 7-O-glucoside) and chlorogenic acid.

Luteolin is a naturally occurring flavonoid compound, distributed in various plant-based foods such as vegetables and fruits, which is recognized for its antioxidant, anti-cancer, anti-inflammatory, and neuro-protective properties [65,66]. It has shown potential biological activity for treating different types of cancer by interacting with various molecular targets and regulating signaling pathways within cancer cells [67]. Luteolin-7-O-glucoside, a glycoside derivative of luteolin, was also observed to be effective against plant pathogens, e.g., *A. alternata*, and human pathogens, e.g., *Enterococcus faecalis*, *Klebsiella pneumoniae*, and *Staphylococcus aureus* [68,69].

Chlorogenic acid is a phenylacrylate polyphenol compound produced in plants through the shikimic acid pathway, for which the biosynthesis involves the enzymatic esterification of caffeic acid (3,4-dihydroxy-cinnamic acid) with quinic acid (1-hydroxyhexahydrogallic acid) [70]. Although further research is essential to elucidate the underlying mechanisms and establish concrete therapeutic applications, it is known for its involvement in cardiovascular health, neuroprotection, and anticancer effects [71].

Although the mechanisms of action of chlorogenic acid were investigated on the human breast cancer cell line MDA-MB231 and it has been concluded that the potentially protective effect of chlorogenic acid does not explain all the properties of artichoke extract, it was therefore hypothesized that the combination of different ubiquitous or specific compounds of artichoke are responsible for the chemopreventive activity [72].

Chlorogenic acid can also act as fungistatic or fungicide when applied at a dose of 15 μg/μL against *Sclerotinia sclerotiorum*, *Fusarium solani*, *Verticillium dahliae*, *Botrytis cinerea*, and *Cercospora sojina* [73]. Shetty et al. [74] showed that silicon application in rose plants induces chlorogenic acid accumulation and that this phenolic acid was at least partially responsible for the reduction of symptoms caused by rose powdery mildew produced by the biotrophic pathogen *Podosphaera pannosa*. In fact, application of chlorogenic acid reduced disease severity by 50%. In addition, Ruelas et al. [75] found that a dose of 50 mm of chlorogenic acid and phenolic acids, compounds extracted from epicarp and mesocarp tissues of tomato, inhibited *A. alternata* spore germination by 30%, whereas the same concentration of caffeic acid and vanillic acid inhibited conidia growth by 16%. The pathogen infection on tomato can also be controlled by treatments with acid phenethyl ester obtaining better results than a commercial fungicide Captan ^®^ (Bayer).

Further experiments are currently underway to elucidate the efficacy of fractions of compounds or single compounds of the artichoke leaf extracts under study to fully understand the mechanism of actions and potential synergistic interactions among the bioactive compounds for their cytotoxic effects on cancer cells and antifungal activity against plant pathogens.

## 4. Material and Methods

### 4.1. Plant Material

Globe artichoke plants were grown at the ex-situ field collection site located in the countryside of Orte municipality (latitude 42°47′ N, longitude 12°33′ E, altitude 130 m). The present study involved the examination of three distinct genotypes. Two of these were chosen as representative genotypes of the two populations identified within the landrace “Carciofo Ortano” [35] and denoted as Orte 1 and Orte 2 genotypes for simplicity. The third genotype, used as a reference, was Grato 1, a clone selected from a progeny derived from cross-pollination of plants belonging to the “Castellammare”, “Campagnano”, and “Violetto di Toscana” landraces. Notably, Grato 1 is included in the varietal platform of the PGI “Romanesco Artichoke of Lazio”.

All artichoke genotypes were vegetatively propagated through offshoots and transplanted into the open field in November 2020, with rows spaced at 1.00 m and inter-row spacing at 2.5 m. The experimental design employed a randomized complete block layout with three replications for each treatment, and each experimental unit comprised five plants. Field experiments were conducted under conditions of limited resource inputs, involving two irrigations per year in April and August, each delivering 60 mm of water. Organic fertilization was performed with 50 kg of nitrogen per hectare, and the cultivation excluded the use of herbicides, pesticides, and gibberellic acid, aligning with the local agronomic practices [35]. After the conclusion of the harvesting period in the 2022 growing season, a minimum of 20 disease-free leaves were harvested from three plants per genotype (one plant per genotype per replicate).

Plant material was immediately sliced and stored at –80 °C, and then freeze-dried. Each lyophilized sample was stored at 4 °C in a sealed plastic bag protected from light and under vacuum until used for analysis. The leaves of nine plants (three genotypes x three biological replicates) were combined, for a total of three samples.

### 4.2. Sample Preparation

Powdered freeze-dried artichoke leaf samples (2.0 g) were extracted with 60 mL of 100% methanol using ultrasound-assisted extraction (200 W) three times (30 min each time) at room temperature. After filtration through 0.45 mm Whatman filter paper to remove all the solids from the extracts, 15.0 mL of each extract was dried using rotavapor under reduced pressure at 30 °C and 20.0 mL of water was added at the solid residue. Purification and fractionation were carried out according to the method described by Schütz et al. [76] with little modification. Briefly, 4.0 mL of aqueous supernatant was loaded to a C18 reversed-phase cartridge previously activated with methanol and rinsed with water and eluted with 10% aqueous MeOH to obtain fraction I (phenolic acids) and then with MeOH to obtain fraction II (neutral compounds). The eluates were evaporated to dryness under vacuum, and the residues obtained were redissolved in 4.0 mL (fraction I) and 1.0 mL (fraction II) of 50% aqueous methanol, respectively.

### 4.3. HPLC-DAD Analysis

Polyphenols were analysed using a Shimadzu “Prominence” system equipped with an SPD-M20A diode array detector. The separation column was a C18 Hyperchrom Bischoff 250 × 4.0 mm i.d., with a 5 μm particle size, thermostated at a temperature of 35 °C. The mobile phase consisted of 2% (*v*/*v*) acetic acid in water (eluent A) and 0.5% acetic acid in water and acetonitrile (50:50, *v*/*v*; eluent B) in the following gradient system (total running time 90 min): initial 10% B, first linear gradient to 18% B in 20 min, second linear gradient to 24% B in 10 min, third linear gradient to 30% B in 15 min, hold at 30% B for 20 min, fourth linear gradient to 55% B in 5 min, final linear gradient to 100% B in 5 min, hold at 100% B for 8 min. The injection volume for all samples was 20.0 μL. Chromatograms were recorded at 320 nm for hydroxy cinnamic acids, at 330 nm for apigenin derivatives, at 350 nm for luteolin derivatives and at 232 nm for cynaropicrin at a flow rate of 0.8 mL/min. The identification of compounds was obtained using retention time and UV spectra comparison of commercial standards. Quantitative analysis was performed according to standard calibration curves of chlorogenic acid for mono-O-caffeoylquinic acids and cynarin for di-O-caffeoylquinic acids, respectively. Apigenin and luteolin derivatives were calculated as apigenin 7-O-glucoside and luteolin 7-O-glucoside, respectively. Cynaropicrin was quantified by the external standard of the commercial cynaropicrin calibration curve. The shown data are the mean of three replicates.

### 4.4. SPME Sampling

With the aim of describing the volatile chemical composition of the samples, sampling was performed using the SPME technique. The applied investigation method followed the one described in our previous works [77,78] further optimized for the examined matrices. In detail, about 2 gr of each sample was placed inside a 7.0 mL glass vial with a PTFE-coated silicone septum. To obtain the better extraction of volatiles, an SPME device from Supelco (Bellefonte, PA, USA) with 1 cm fiber coated with 50/30μm DVB/CAR/PDMS (divinylbenzene/carboxen/polydimethylsiloxane) was used. Before use, the fiber was conditioned at 270 °C for 30 min. The equilibration time for all samples was obtained heating to 40 °C for 10 min. After this time, the fiber was exposed to the headspace of the samples for 30 min at 40 °C to capture and concentrate the volatile molecules. Lastly, the analytes were desorbed thermally in the GC injector maintained at 250 °C for 2 min in splitless mode.

### 4.5. GC-MS Analysis of Samples

The analyses were carried out on a Clarus 500 model Perkin Elmer (Waltham, MA, USA) gas chromatograph equipped with FID (flame detector ionization) and coupled with a mass spectrometer. To obtain the separation of detected components, a capillary column Varian Factor Four VF-1 was used. The programmed oven temperature was set as follows: it was held at 50 °C and then programmed at 6 °C/min to 220 °C and held for 10 min. Helium was used as carrier gas at a constant rate of 1.0 mL/min. The MS conditions were the following: ion source, 180 °C; electron energy, 70 eV; quadrupole temperature, 200 °C; GC-MS interface zone, 220 °C; scan range, 35–450 mass units. The identification of compounds was based on the comparison of the mass spectra of pure components stored in the Wiley 2.2 and Nist 02 libraries database and on the comparison of the linear retention indices (LRIs) calculated using a series of alkane standards (C_8_–C_25_ n-alkanes) with the available retention data reported in the literature. The relative amounts of the identified molecules were expressed as percentages and were obtained using FID peak-area normalization (mean of three replicates) without the use of an internal standard and any factor correction.

### 4.6. Antiproliferative and Cytotoxic Activities

Human neuroblastoma cells (SH-SY5Y, ATCC^®^ CRL-2266), human adenocarcinoma breast cells (MCF7, ATCC^®^ HTB-22 and MDA, ATCC^®^ MB-231) and normal breast epithelial cells (MCF10A, ATCC^®^ CRL-10317) were used to determine the antiproliferative and cytotoxic activity of the “Carciofo Ortano” leaf extracts.

Neuroblastoma and adenocarcinoma cells were cultured in DMEM F-12 supplemented with 10% fetal bovine serum, 1% glutamine and 1% penicillin–streptomycin. The MCF10A cells were maintained in DMEM F-12 medium supplemented with 100 ng/mL cholera toxin, 20 ng/mL epidermal growth factor (EGF), 0.01 mg/mL insulin, 500 ng/mL hydrocortisone, 5% horse serum (HS), 1% glutamine and 1% penicillin–streptomycin. Before being seeded for the viability assay, the culture medium of the normal cells was deprived of EGF and the HS was reduced to 2%.

The cells were maintained at 37 °C and in 5% CO_2_ until the logarithmic phase of growth was reached and then used for the analyses.

The MTT assay was performed using the protocol described by Laghezza Masci and colleagues [79]. At 24 h after seeding, the cells were treated with ten two-fold concentrations, from 250 to 0.48 µg/mL of extracts dissolved in dimethylsulphoxide (DMSO). The cells without treatment and treated with DMSO were used as the negative and solvent control, respectively.

The antiproliferative activity of the samples was investigated in a dose- and time-dependent manner for 24 h, 48 h and 72 h while, to assess the cytotoxicity effect, the cells were treated for 72 h in dose-independent mode. After each incubation time, the treatments were removed and 100 µL of MTT (0.5 mg/mL) was added and incubated for 3 h at 37 °C and 5% of CO_2_ until formazane crystals were completely formed. The absorbance was read at 595 nm after the addition of 100 µL of DMSO.

The half-maximal concentration (EC_50_) for each biological replicate was determined using a logical response curve constructed using GraphPad Prism version 8.0.2 for Windows (GraphPad Software, San Diego, CA, USA). Three independent biological tests were used to calculate the overall EC_50_.

### 4.7. Antibacterial Activities

*B. cereus* (ATCC 10.876, Gram+), *K. marina* (DSM 16.420, Gram+), *A. bohemicus* (DSM 102.855, Gram+) and *E. coli* (ATCC 25.922, Gram-) and *P. fluorescens* (ATCC 13.525, Gram-) bacterial strains were used to evaluate the antibacterial activity of the extracts.

Lysogeny Broth agar containing 10 g/L tryptone, 5 g/L yeast extract, 10 g/L NaCl, and 15 g/L agar was sterilized and used to maintain all the bacterial strains at 26 °C for *B. cereus*, *A. bohemicus* and *P. fluorescens*, and 37 °C for *K. marina* and *E. coli*. Fresh culture plates inoculated with each different strain were prepared the day before the analysis.

The MIC was determined according to the microdilution method [80].

Each bacterial strain suspension (10^6^ CFU/mL) was treated by adding 12 dilutions of extracts in LB (from 4 mg/mL to 0.002 mg/mL). Negative, positive, and solvent controls were obtained using medium, medium with gentamicin (from 100 µg/mL to 0.05 µg/mL) and medium with DMSO (from 5% to 0.002%), respectively. After 24 h of incubation, 10 µL of MTT (0.5 mg/mL) was added to visualize bacterial growth. Each assay was repeated in triplicate. After 24 h, the MBC was determined for the dilution samples in which no bacterial growth was observed.

### 4.8. Antifungal Activities

Stock solution of extracts obtained from the leaves of three artichoke accessions as described above, Orte 1, Orte 2 and Grato 1, was prepared in dimethylsulfoxide (DMSO). Screening for antifungal activity of the extracts was performed qualitatively using the microdilution broth assay against a saprophytic fungus, namely *Alternaria alternata* Mn2, obtained from the Plant Protection Laboratory of the DIBAF department (University of Tuscia, Viterbo, Italy). Extracts were tested at concentrations of 0.56, 1.13, 2.25, 3.38 and 4.5 mg/mL.

In order to obtain conidia, *A. alternata* Mn2 was cultured on PDA (Potato Dextrose Agar, VWR Chemicals prolabo, Radnor, PA, USA, 39 g/L) in 9 cm petri dishes at 24 °C for 7–10 days. Harvesting was carried out by suspending the conidia in sterile water. The spore suspension was then filtered and transferred into tubes, and the concentration was adjusted to 5 × 10^4^ conidia/mL. The determination of minimum inhibitory concentrations (MICs) was performed with a serial dilution technique using 96-well microtiter plates. The extracts investigated were dissolved in potato dextrose broth (PDB, VWR Chemicals prolabo, 24 g/L) with *A. alternata* Mn2 inoculum (5 × 10^4^ conidia/mL). The microplates were incubated for 4 h at 24 °C. The activity was determined by counting conidia germination using a Zeiss Axioskop 50 microscope. PDB medium and PDB + DMSO were used as controls.

### 4.9. Statistical Analysis

Statistical analysis was performed using one-way analysis of variance (ANOVA) followed by Tukey multiple comparisons (*p* < 0.05). The software Graph Pad Prism (version 8.0.1 for Windows, GraphPad Software, San Diego, CA, USA) was used for data analysis.

## 5. Conclusions

This paper proposes a sustainable approach for re-use of artichoke waste, offering an alternative to conventional methods like discharge. The study demonstrated that by-products derived from artichoke leaves can serve as a source of bioactive compounds for use in medicine and agriculture. Our study’s findings are consistent with those from literature, indicating that phenol-rich extracts from artichoke leaves can have both anticancer and antimicrobial activities. Moreover, according to the authors’ knowledge, this study is the first to report on the antifungal activity of artichoke leaf extracts against *A. alternata*.

Additional investigations are required to ascertain the optimal dosage and formulation for maximizing the efficacy of bioactive compounds found in artichoke leaves, as well as for elucidating the mechanisms of action and potential synergies concerning their cytotoxic effects and antimicrobial activities.

## Figures and Tables

**Table 1:** Amount of phenols and cynaropicrin (mean value ± SD) in Orte 1, Orte 2 and Grato 1 leaf extract (mg/kg DW) (n.d.: not detected). Different letters (a, b, c) indicate statistically significant differences at *p* < 0.05.

	Orte 1	Orte 2	Grato 1
1-O-caffeoylquinic acid	106.8 ± 2.8	96.3 ± 20.6	59.5 ± 1.6
3-O-caffeoylquinic acid	141.9 ± 1.0	156.0 ± 10.1	59.7 ± 0.6
5-O-caffeoylquinic acid (chlorogenic acid)	5156.7 ± 11.0	7842.9 ± 430.7	2425.2 ± 39.3
Caffeic acid	238.0 ± 3.6	118.4 ± 3.2	34.0 ± 0.4
1,3-di-O-caffeoylquinic acid (cynarin)	n.d.	67.1 ± 8.5	51.8 ± 1.1
3,5-di-O-caffeoylquinic acid	385.7 ± 4.6	2970.9 ± 238.3	305.1 ± 31.1
1,5-di-O-caffeoylquinic acid	1750.7 ± 71.1	4133.1 ± 96.1	696.2 ± 74.9
**total caffeoylquinic acids**	**7780.0 ± 88.6 b**	**15,384.9 ± 263.0 a**	**3631.4 ± 58.9 c**
luteolin 7-O-rutinoside	994.5 ± 1.6	665.3 ± 8.7	1197.9 ± 116.5
luteolin 7-O-glucoside	1591.5 ± 13.6	764.7 ± 18.1	2036.5 ± 43.0
luteolin	n.d.	n.d.	10.8 ± 1.2
**total luteolin**	**2586.0 ± 15.3 b**	**1429.9 ± 26.8 c**	**3245.2 ± 160.8 a**
apigenin 7-O-glucoside	n.d.	n.d.	5.8 ± 0.5
apigenin	n.d.	n.d.	n.d.
**total apigenin**	**n.d.**	**n.d.**	**5.8 ± 0.5**
			
**total polyphenols**	**10,366.0 ± 73.4 b**	**16,814.8 ± 236.2 a**	**6882.4 ± 220.1 b**
			
Not identified	283.4 ± 6.1	n.d.	495.4 ± 30.9
cynaropicrin	3946.3 ± 5.4a	8143.3 ± 2845.4a	4994.9 ± 253.7a

**Table 2:** Chemical volatile composition (percentage mean value ± SD) of Orte 1, Orte 2, and Grato 1 fresh leaves.

Components ^1^	LRI ^2^	LRI ^3^	Orte 1	Orte 2	Grato 1
3-methyl-1-butanol	699	700	39.9 ± 3.6	71.4 ± 9.5	73.4 ± 10.1
2-methyl-1-butanol	748	744	21.0 ± 2.1	28.4 ± 0.1	26.6 ± 5.1
3-hexen-1-ol	868	875	39.1 ± 4.1	n.d.	n.d.
β-caryophyllene	1414	1418	n.d.	0.1 ± 0.0	n.d.
β-eudesmene	1488	1481	n.d.	0.1 ± 0.0	n.d.

^1^ The components are reported according to their elution order on apolar column; ^2^ linear retention indices measured on apolar column; ^3^ linear retention indices from literature; n.d. not detected.

**Table 3:** EC_50_ values of Orte 1, Orte 2 and Grato 1 leaf extract on the different cell lines considered. The values are expressed as mean value ± SD and the EC_50_ concentration reported in μg/mL.

		Orte 1	Orte 2	Grato 1
SH-SY5Y	24 h	18.3 ± 0.2	51.8 ± 5.3	16.9 ± 0.6
	48 h	12.6 ± 0.5	47.1 ± 9.1	21.9 ± 0.5
	72 h	35.4 ± 1.2	59.8 ± 3.6	33.7 ± 0.8
MCF7	24 h	85.2 ± 2.3	>250	46.2 ± 1.8
	48 h	44.7 ± 4.2	95.0 ± 2.3	77.1 ± 3.4
	72 h	23.3 ± 1.2	33.6 ± 1.6	35.6 ± 1.6
MDA	24 h	69.0 ± 2.3	75.6 ± 1.8	49.2 ± 0.5
	48 h	33.4 ± 0.9	46.3 ± 3.2	14.3 ± 1.9
	72 h	26.5 ± 1.2	40.1± 2.9	17.7 ± 0.4
MCF10A	24 h	>250	>250	>250
	48 h	>250	>250	>250
	72 h	>250	>250	>250

**Table 4:** MIC and MCB values of Orte 1, Orte 2 and Grato 1 leaf extract on the different bacterial strains considered. The values are reported in mg/mL. (n.d.: not detected).

	*B. cereus*	*K. marina*	*A. bohemicus*	*E. coli*	*P. fluorescens*
	**Orte 1**				
MIC	0.5	n.d.	2.0	n.d.	n.d.
MBC	1.0	n.d.	4.0	n.d.	n.d.
MIC/MBC	0.5	n.d.	0.5	n.d.	n.d.
	**Orte 2**				
MIC	2.0	n.d.	2.0	n.d.	n.d.
MBC	2.0	n.d.	2.0	n.d.	n.d.
MIC/MBC	1.0	n.d.	1.0	n.d.	n.d.
	**Grato 1**				
MIC	2.0	n.d.	2.0	n.d.	n.d.
MBC	2.0	n.d.	4.0	n.d.	n.d.
MIC/MBC	1.0	n.d.	0.5	n.d.	n.d.

## Data Availability

The data contained within the present article and in its Supplementary Materials are freely available upon request to the corresponding author.

## Supplementary Materials

The following supporting information can be downloaded at: https://www.mdpi.com/article/10.3390/plants13060761/s1, Figure S1: Representative HPLC-DAD profile of caffeoylquinic acids [fraction I (A)] and flavones and cynarin [fraction II (B)] in “Carciofo Ortano” leaves [320 nm (A) and 245 nm (B)]. Assigned peaks numbers follow those listed in Table S1.; Table S1: Phenolic compounds detected in “Carciofo Ortano” leaves: compound number, chemical structure, retention time, and detection wavelength.
