# Supplementary material for: Cynara cardunculus L. var. scolymus L. Landrace “Carciofo Ortano” as a Source of Bioactive Compounds
Source: Plants (Basel). 2024 Mar 7;13(6):761. doi: 10.3390/plants13060761 (PMC10976138; doi:10.3390/plants13060761)
Supplement: Supplementary file 1 [file plants-13-00761-s001.zip › plants-2885836-supplementary.pdf]

Supplementary materials

A

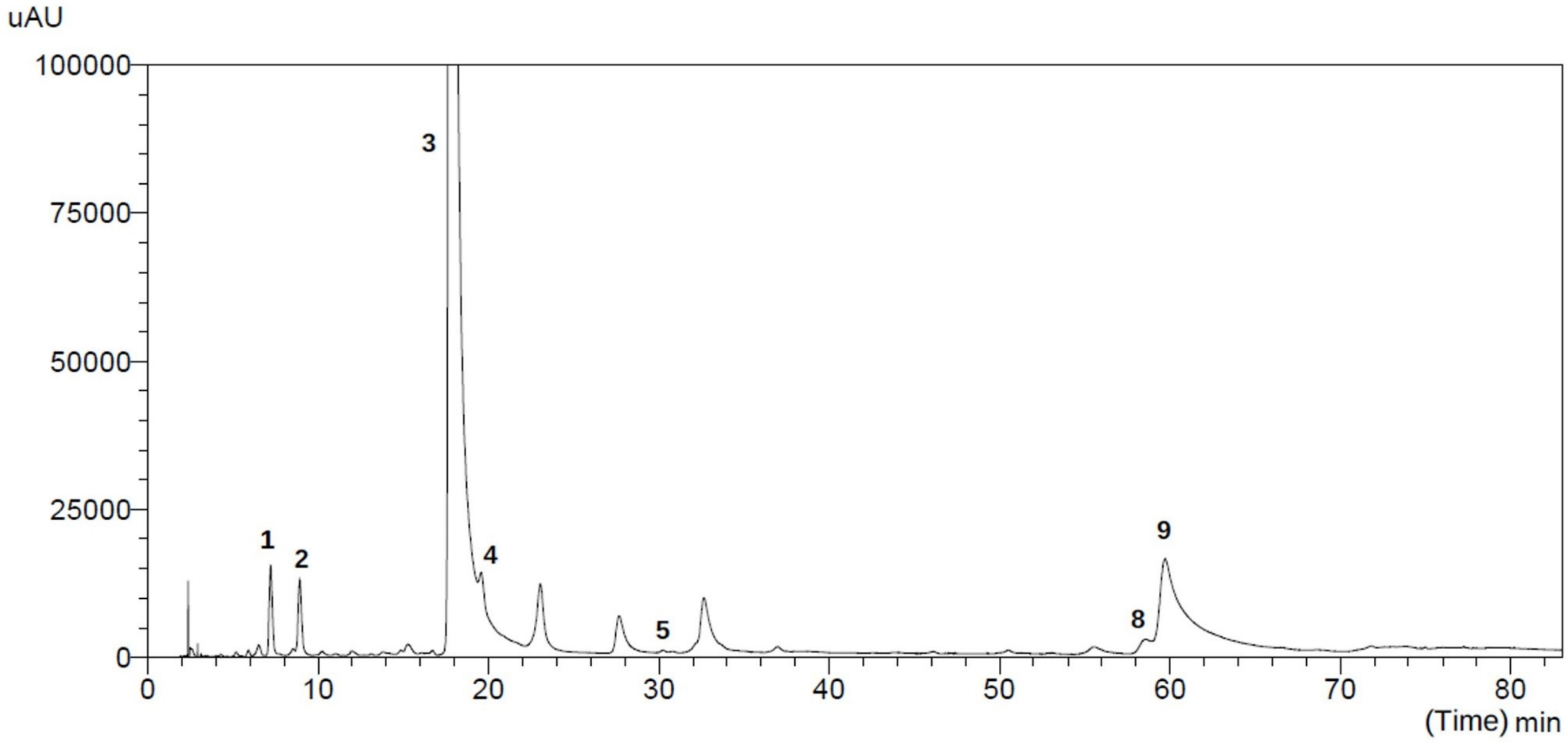

B

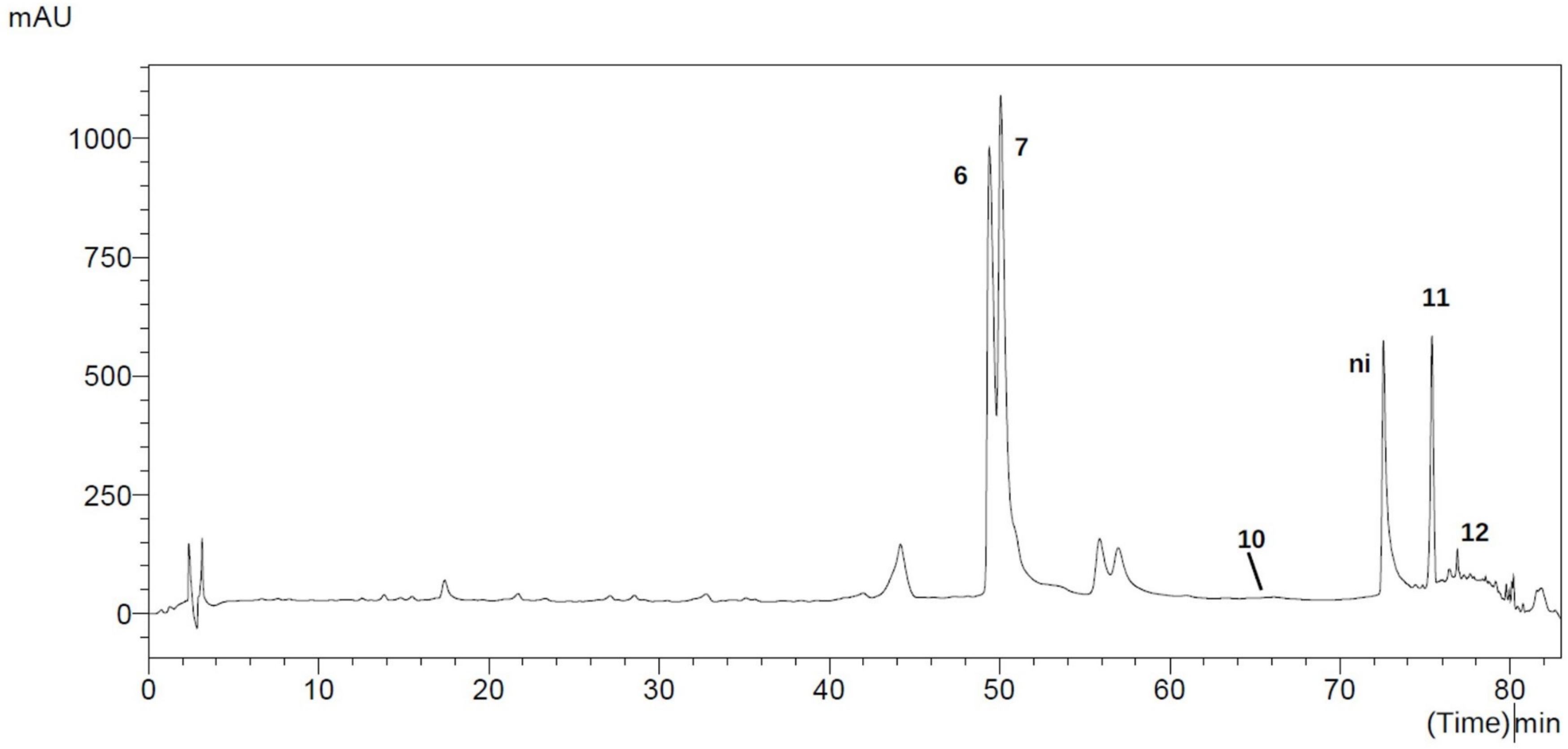

Figure S1. Representative HPLC-DAD profile of caffeoylquinic acids [fraction I (A)] and flavones and cynarin [fraction II (B)] in "Carciofo Ortano" leaves [320 nm (A) and 245 nm (B)]. Assigned peaks numbers follow those listed in Table S1.

Table S1. Phenolic compounds detected in “Carciofo Ortano” leaves: compound number, chemical structure, retention time, and detection wavelength.

Compound No	Name	Chemical Structure	Retention time (min)	Detection Wavelength (λ, nm)
1	1-O-caffeoylquinic acid	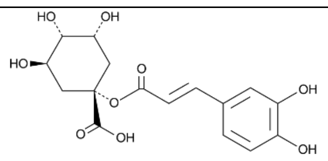	7.21	320
2	3-O- caffeoylquinic acid	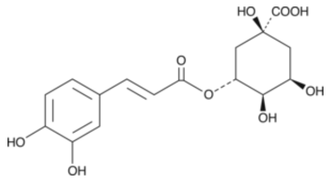	9.05	320
3	5-O- caffeoylquinic acid	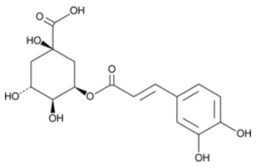	17.84	320
4	caffeic acid	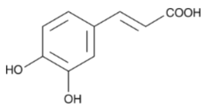	19.95	320
5	1,3-di-caffeoylquinic acid (cynarin)	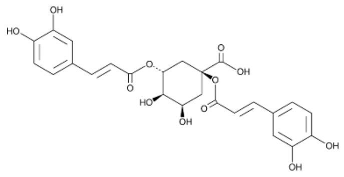	30.15	320
6	luteolin 7-O-rutinoside	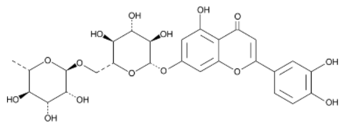	49.82	350
7	luteolin 7-O-glucoside	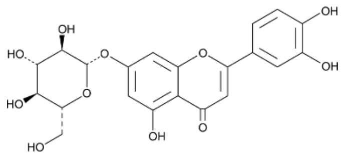	50.79	350
8	3,5-di-O-caffeoylquinic acid	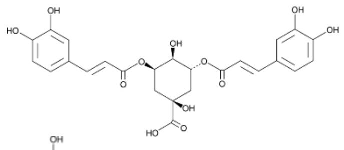	58.67	320
9	1,5-di-O-caffeoylquinic acid	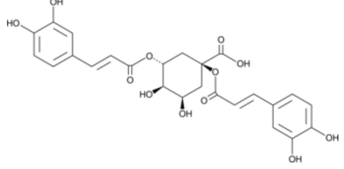	59.93	320
10	apigenin 7-O-glucoside	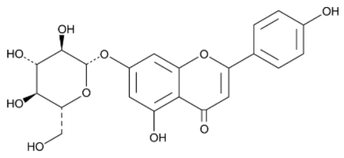	66.09	330
11	not identified		73.05	320
12	cynaropicrin	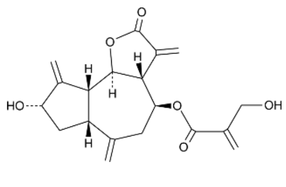	75.83	232
13	luteolin	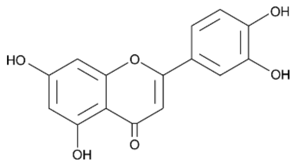	76.81	350
